# Personal Matters

**Published:** 1856-05

**Authors:** 


### EDITORIAL.

*Personal Matters.*

The editor of the *Buffalo Medical Journal*, in his April number, closes an allusion to the *Peninsula Journal* and our recent controversy with one of its editors, with the following paragraph:—

"In that hope, we shall say nothing more at this time—except to express our wonder at the very pleasant manner in which the *Peninsular* is soothing its quandom antagonist, the *North-Western*. All through the stormy fall and winter months, the *Peninsular* has showered down epithets on the *North-Western*, implying every sin in the decalogue (though dwelling particularly on the *eighth* commandment;) but now the *pleasant May is coming*, and the *Peninsular* is billing and cooing towards the *North-Western* like any turtle dove! What does it mean?"

Sure enough, what does it mean? Why, friend Hunt, it means simply, that it is more in accordance with the tactics and sense of propriety of the *Peninsular* to commence playing the part of the dove, all at once, than to give its readers a manly and honorable reply to the demands made upon it in the December number of this journal.

It may not be amiss to inform our readers that the only reply which the editor of the *Peninsular Journal* has made to those demands, is contained in the following paragraph from the March number of that journal:—

"As that contest, which was originally confined to the representatives of the rival schools, Professors Palmer and Davis, was sometime since terminated by the assumption of *a sort of armed neutrality on the part of the Journals*, through which they had spoken, we think the recent allusion of the *Buffalo Journal* to the subject, is a mere manœuvre on the part of the editor, by which he may retreat from the field himself, under cover of the smoke from our batteries, which had already ceased firing."

Now, we are perfectly willing that those who control the *Peninsular Journal* should end the controversies of any one of its editors, by making it "assume a sort of armed neutrality." Such a course may be convenient sometimes, to relieve its editorial corps from difficulties; but we very respectfully object to their including *our Journal* under *their* supervision. In using the plural number, in the above quotation, they have *kindly*, no doubt, informed their readers, that this Journal has also "assumed a sort of armed neutrality." We take this opportunity, however, to give our Michigan neighbors due notice, that the *North-Western Medical and Surgical Journal* is under neither dictation nor ownership aside from its editors —that it has assumed no sort of "armed neutrality" on any subject; and, further, that its editors belong neither to the *neutral* nor *neuter genders.*

